# Which role for nitric oxide in symbiotic N_2_-fixing nodules: toxic by-product or useful signaling/metabolic intermediate?

**DOI:** 10.3389/fpls.2013.00384

**Published:** 2013-10-09

**Authors:** Alexandre Boscari, Eliane Meilhoc, Claude Castella, Claude Bruand, Alain Puppo, Renaud Brouquisse

**Affiliations:** ^1^Institut National de la Recherche Agronomique, Institut Sophia Agrobiotech, UMR 1355Sophia Antipolis, France; ^2^Centre National de la Recherche Scientifique, Institut Sophia Agrobiotech, UMR 7254Sophia Antipolis, France; ^3^Institut Sophia Agrobiotech, Université Nice Sophia AntipolisSophia Antipolis, France; ^4^Institut National de la Recherche Agronomique, Laboratoire des Interactions Plantes-Microorganismes, UMR441Castanet-Tolosan, France; ^5^Centre National de la Recherche Scientifique, Laboratoire des Interactions Plantes-Microorganismes, UMR2594Castanet-Tolosan, France

**Keywords:** legume, nitric oxide, nitrogen fixation, rhizobium, symbiosis

## Abstract

The interaction between legumes and rhizobia leads to the establishment of a symbiotic relationship characterized by the formation of new organs called nodules, in which bacteria have the ability to fix atmospheric nitrogen (N_2_) via the nitrogenase activity. Significant nitric oxide (NO) production was evidenced in the N_2_-fixing nodules suggesting that it may impact the symbiotic process. Indeed, NO was shown to be a potent inhibitor of nitrogenase activity and symbiotic N_2_ fixation. It has also been shown that NO production is increased in hypoxic nodules and this production was supposed to be linked – via a nitrate/NO respiration process – with improved capacity of the nodules to maintain their energy status under hypoxic conditions. Other data suggest that NO might be a developmental signal involved in the induction of nodule senescence. Hence, the questions were raised of the toxic effects versus signaling/metabolic functions of NO, and of the regulation of NO levels compatible with nitrogenase activity. The present review analyses the different roles of NO in functioning nodules, and discusses the role of plant and bacterial (flavo)hemoglobins in the control of NO level in nodules.

## INTRODUCTION

Nitric oxide (NO) is a gaseous molecule which was found to be involved in plant development, and response to biotic or abiotic stresses (Besson-Bard et al., 2008). NO production was also reported during symbiotic interactions, particularly in the nitrogen (N_2_)-fixing symbiosis (NFS) between legumes and soil Gram-negative bacteria called rhizobia (Baudouin et al., 2006). The interaction between legumes and rhizobia leads to the establishment of a symbiotic relationship characterized by the formation of new differentiated organs called nodules, which provide a niche for bacterial N_2_ fixation. In the nodules, bacteria released in plant cells differentiate into bacteroids with the ability to fix atmospheric N_2_ via nitrogenase activity (Oldroyd and Downie, 2008). As nitrogenase is strongly inhibited by oxygen, N_2_ fixation requires the microaerophilic conditions prevailing in the nodules (Appleby, 1992). Thus, nodule development occurs in changing oxygen conditions, shifting from a normoxic environment during symbiosis establishment to a microoxic one in functioning nodules. During the last decade, increasing evidence of the presence of NO during symbiosis, from early interaction steps between the plant and the bacterial partners to N_2_-fixing and senescence steps in mature nodules, has been reported (for review, see Meilhoc et al., 2011). At later stages of the interaction, NO was observed to be produced in N_2_-fixing nodules of *Medicago truncatula* and *M. sativa* particularly in bacteroid-containing cells (Baudouin et al., 2006; Pii et al., 2007). NO was also detected directly in mature nodules of *Lotus japonicus* (Shimoda et al., 2009), and indirectly through the detection of nitrosylleghemoglobin complexes in nodules of soybean and pea (Kanayama et al., 1990; Mathieu et al., 1998; Meakin et al., 2007). Interestingly, both the plant and the bacterial partners were shown to participate significantly in NO synthesis (Sanchez et al., 2010; Horchani et al., 2011).

The chemical nature, concentration, and location of NO might influence its biological role, and at high local concentration NO can become very toxic. NO was thus shown to inhibit the growth of *Sinorhizobium meliloti* in culture (Meilhoc et al., 2010), and the symbiotic N_2_ fixation in legumes (Sasakura et al., 2006; Shimoda et al., 2009; Kato et al., 2010). However, more recently NO has been found to play a beneficial metabolic function for the maintenance of the energy status (Horchani et al., 2011), or to have a regulatory role in the regulation of N_2_ metabolism (Melo et al., 2011) in functioning nodules. These observations raised the question of the role of NO in N_2_-fixing nodules. This review focuses on the toxic versus metabolic roles of NO in symbiotic nodules, and discusses the role of plant and bacterial hemoglobins (Hbs) in the control of NO levels in nodules.

## THE PRODUCTION OF NO IN NODULES

**FIGURE 1:**
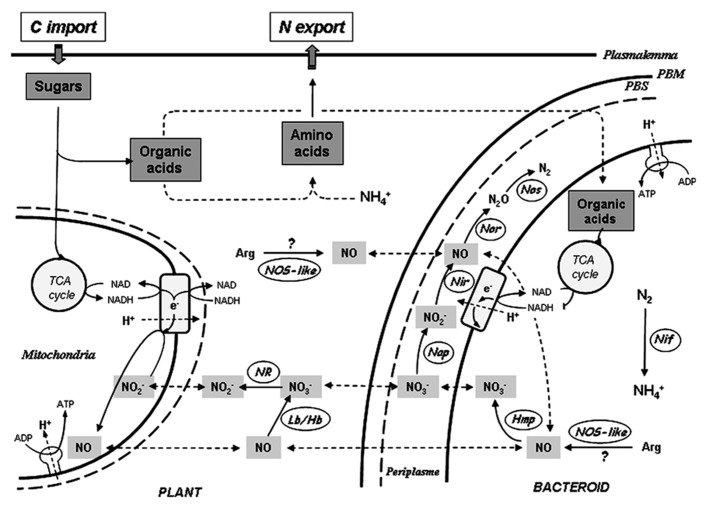
**Schematic representation of NO sources in nitrogen-fixing nodules.** On the plant side, NO is produced through the cyclic nitrate–NO respiration pathway involving nitrate reductase (NR), mitochondrial electron transfer chain (ETC) and hemoglobin (Lb/Hb). The production of NO via a plant NO synthase-like enzyme (NOS-like) is hypothetical. On the bacteroid side, NO is produced as an intermediate of the denitrification pathway involving nitrate reductase (Nap), nitrite reductase (Nir), NO reductase (Nor), and nitrous oxide reductase (Nos). In the cytosol NO is oxidized into ${NO}_{3}^{-}$ by the flavohemoglobin Hmp. The production of NO via a bacterial NOS-like is hypothetical. In both plant and bacteroid partners, ATP is synthesized due to transmembrane electrochemical gradient generated by proton (H^+^) pumping at ETC level. ${NO}_{3}^{-}$, nitrate; ${NO}_{2}^{-}$, nitrite; N_2_O, nitrous oxide; ${NH}_{4}^{+}$, ammonium; Arg, arginine; Nif, nitrogenase; PBM, peribacteroid membrane; PBS, peribacteroid space.

The origin of NO in NFS is still unclear and several NO sources have been evidenced (**Figure 1**). Some studies argue in favor of the involvement of a NO synthase (NOS)-like enzyme. Thus, in *Lupinus albus* nodule extracts, NO and L-[^14^C] citrulline were found to be produced in an L-arginine-dependent manner, and the production of L-citrulline was inhibited by a NOS inhibitor (*N*^ω^-monomethyl-L-arginine, L-NMMA; Cueto et al., 1996). Baudouin et al. (2006) showed that the addition of L-NMMA in *M. truncatula*–*S. meliloti* nodule slices impaired NO detection. More recently, the growth and viability of soybean – *Bradyrhizobium japonicum* nodules was found to be negatively affected by the NOS inhibitor *N*^ω^-nitro-L-arginine (L-NNA; Leach et al., 2010). However, the molecular identity of such a NOS-like enzyme remains unknown. Using both pharmacological and genetic approaches, Horchani et al. (2011) addressed the role of plant nitrate reductase (NR) and mitochondrial electron transfer chain (ETC) in NO production in *M. truncatula–S. meliloti* nodules. NO production was thus found to be inhibited by tungstate (Tg), a NR inhibitor. In addition, nodules obtained with plant NR RNA-interference (RNAi) double knockdown (MtNR1/2) exhibited reduced NR activities and NO production levels. The reduction of NO production was reversed by nitrite addition, both in the Tg-treated nodules and in MtNR1/2 RNAi nodules, indicating that NO synthesis depends on NR activity, but that NR does not produce NO directly. The inhibition of NO production by ETC inhibitors indicated that mitochondrial ETC was the site of nitrite reduction into NO (Horchani et al., 2011). Thus, in *M. truncatula* nodules, nitrate may be reduced into NO in a two-step mechanism involving successively NR and ETC.

In rhizobia, the denitrification pathway depends on the *napEDABC*, *nirKV*, *norCBQD*, and *nosRZDYFLX* genes that encode NR, nitrite reductase (NiR), NO reductase (Nor), and nitrous oxide (N_2_O) reductase, respectively (Bedmar et al., 2005). The expression of the denitrification genes *nirK, norC*, and *nosZ* has been reported in soybean – *B. japonicum* functional nodules (Mesa et al., 2004). Using *B. japonicum napA* and *nirK* mutants, it was shown that bacteroid NR and NiR contribute to the main part of NO production, particularly under hypoxic conditions (Meakin et al., 2007; Sanchez et al., 2010). Using a genetic approach, Horchani et al. (2011) similarly showed that around one-third of the NO generated by *M. truncatula–S. meliloti* nodules is produced via the bacteroid denitrification pathway. To date, although a L-arginine-dependent NO synthesis has been reported in free-living *S. meliloti* cells (Pii et al., 2007), such a production was not described in functioning nodules.

## NO: THE FOX TO MIND THE GEESE

Nitric oxide concentration was roughly estimated to be in the micromolar range in *Medicago* nodules (Meilhoc et al., 2010), and its level was significantly increased under hypoxic conditions or when nitrate was applied to nodules (Kato et al., 2010; Sanchez et al., 2010; Horchani et al., 2011). NO was first reported to be a potent inhibitor of the *B. japonicum* nitrogenase activity, with a Ki of 56 μM (Trinchant and Rigaud, 1982). The addition of NO donors to *Lotus* and *Alnus firma* nodules, although probably exceeding *in vivo* NO concentrations, led to a reduction in N_2_ fixation efficiency (Sasakura et al., 2006; Shimoda et al., 2009; Kato et al., 2010). In this context, *M. truncatula* inoculated with a *S. meliloti* *hmp* (a bacterial NO-scavenging flavohemoglobin, f-Hb) mutant affected in NO degradation, exhibited a higher NO content in the nodules and a reduced N_2_ fixation efficiency as compared to the wild type (WT) strain (Cam et al., 2012). Such effects were indirectly confirmed in *Lotus japonicus* nodules, where the over-expression of non-symbiotic Hb (ns-Hb1, a NO-scavenging enzyme), led to increased N_2_ fixation efficiency (Shimoda et al., 2009).

**FIGURE 2:**
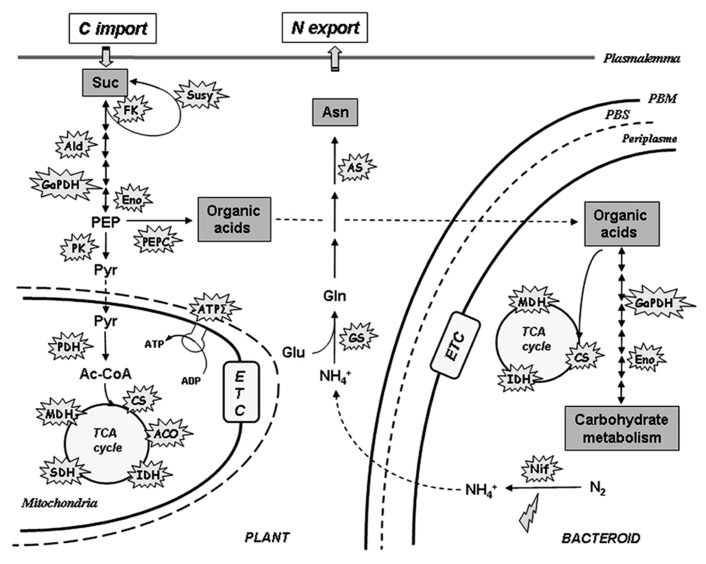
**Schematic representation of NO targets in nitrogen, carbon, and energy metabolism in nitrogen-fixing nodules.** Explosions refer to enzymes from both plant and bacterial partners found to be either *S*-nitrosylated, or/and inhibited, by NO. Lightning refers to gene repression by NO. Ac-CoA, acetyl-CoA; Asn, asparagine; Glu, glutamate; Gln, glutamine; ${NH}_{4}^{+}$, ammonium; PEP, phosphoenolpyruvate; Pyr, pyruvate; Suc, sucrose. Aco, aconitase; Ald, aldolase; AS, asparagine synthetase; ATPΣ, ATP synthase; CS, citrate synthase; Eno, enolase; ETC, electron transfer chain; FK, fructokinase; GaPDH, glyceraldehyde-3-phosphate dehydrogenase; GS, glutamine synthetase; IDH, isocitrate dehydrogenase; MDH, malate dehydrogenase; Nif, nitrogenase; PEPC, PEP carboxylase; PDH, pyruvate dehydrogenase; PK, pyruvate kinase; SDH, succinate dehydrogenase; Susy, sucrose synthase; PBM, peribacteroid membrane; PBS, peribacteroid space.

Nitric oxide is known to modify proteins through *S*-nitrosylation, which emerges as a key post-translational modification in plants and a pivotal mechanism to mediate NO bioactivity (Astier et al., 2012). Nitrogenase displays at least three putative *S*-nitrosylation sites (Xue et al., 2010) and, interestingly, different nitrogenase subunits were identified among the *S*-nitrosylated proteins found in *M. truncatula* mature nodules (Puppo et al., 2013). This suggests that NO may inhibit nitrogenase activity through *S*-nitrosylation. Moreover, it was demonstrated that in soybean nodules the NO produced in response to flooding decreased the expression of *B. japonicum* *nifH* and *nifD* genes encoding the Fe protein and the α-subunit of the MoFe protein of nitrogenase respectively (Sanchez et al., 2010). These observations indicate that at both transcriptional and post-translational levels nitrogenase appears as a primary target for the inhibition of N_2_ fixation by NO (**Figure 2**).

Using a *S. meliloti hmp* mutant, Cam et al. (2012) recently showed that an increase in the NO level within the nodule causes its premature senescence, whereas over-expression of *hmp* in nodules leads to a significant delay in nodule senescence, and partly relieves dark-induced senescence of the nodules. These results and others (Cam et al., 2012) provide evidence that NO is produced during aging of legume nodules, and suggest that it could stimulate the senescence of nodules.

## NO: A RESPONSE TO HYPOXIA

Based on known adaptation mechanisms of plants to hypoxia, and considering that nodules are microoxic organs, a metabolic role for NO in functioning nodules has been recently proposed (Horchani et al., 2011; Meilhoc et al., 2011). NO production is induced in the roots of plants submitted to hypoxia, and this production is supposed to be linked – via a cyclic respiration process – with improved capacity of the plants to cope with hypoxic stress and to maintain cell energy status (Igamberdiev and Hill, 2009; Gupta and Igamberdiev, 2011). This cyclic respiration, called “nitrate–NO respiration,” involves four successive steps (**Figure 1**): (1) the reduction of nitrate to nitrite by NR, (2) the translocation of nitrite from the cytosol into the mitochondria, (3) the reduction of nitrite in NO, via the mitochondrial ETC, allowing respiration and ATP regeneration, and (4) the diffusion of NO from the matrix to the cytosol, where it is oxidized in nitrate by ns-Hb. Thus, under hypoxic conditions, by reducing nitrite to NO, plant mitochondria preserve the capacity to oxidize external NADH, and retain a limited power for ATP synthesis complementing glycolytic ATP production (Gupta and Igamberdiev, 2011).

In functional nodules of *G. max* (Meakin et al., 2007) and *M. truncatula* (Horchani et al., 2011) NO production is increased under hypoxic conditions, and several observations argue in favor of the involvement of nitrate–NO respiration in nodule energy supply. First, plant NR and ETC, and the bacterial denitrification pathway contribute to NO production, via nitrate and nitrite reduction, particularly under hypoxic conditions (Sanchez et al., 2010; Horchani et al., 2011). Second, leghemoglobins (Lbs) and ns-Hb have the capacity to efficiently react with NO to produce nitrate with an elevated rate constant (Herold and Puppo, 2005), and the NO generated at the ETC level may therefore be oxidized into nitrate by Lbs and/or ns-Hbs. Third, the energy status of the nodules depends either partly, or almost entirely, on NR functioning under normoxic, or hypoxic conditions, respectively (Horchani et al., 2011). Thus, in symbiotic nodules a role related to NO metabolism may be fulfilled by Hbs and Hmp in the plant and bacterial partner respectively. The high affinity of these Hbs for NO and their capacity to oxidize NO into nitrate would be favorable to supply the nitrate–NO respiratory cycle in order to maintain a minimal energy status under hypoxia.

On the other hand, during the N_2_ fixing process, ammonium generated by bacteroid nitrogenase activity and released in the cytosol of plant cells, is assimilated trough the plant glutamine synthetase (GS1) activity. It has been shown that the *M. truncatula* cytosolic GS1 activity is modulated by NO-mediated tyrosine nitration (Melo et al., 2011). According to the model proposed by the authors, the inhibition of GS1 activity by tyrosine nitration could be directly related to the NO-induced nitrogenase inhibition and the subsequent decrease in ammonium level. Interestingly, a recent analysis of *M. truncatula*–*S. meliloti* nodules resulted in the identification of about 80 *S*-nitrosylated proteins, such as enzymes of the tricarboxylic acid (TCA) cycle, glycolysis, and N_2_ assimilation from either the plant or the bacterial partner (**Figure 2**; Puppo et al., 2013). The activity of some of these enzymes was also found to be inhibited by NO donors (Brouquisse and Castella, unpublished). Considered together, these data suggest that in nodules, NO could also function as a down-regulator of N_2_-fixation and carbon metabolism to reduce energy demand under strong hypoxic conditions (**Figure 2**).

## ROLE OF HEMOPROTEINS IN THE CONTROL OF NO LEVEL

Toxic, signaling, or metabolic effects of NO depend on its concentration at the site of action (Mur et al., 2012). Thus, in *Lotus japonicus* nodules, high concentrations of NO inhibit N_2_ fixation, while low concentrations of NO enhance it (Kato et al., 2010). Therefore, NO steady-state concentration inside nodules should be tightly controlled to limit toxic effects and allow the signaling and metabolic function(s) to occur.

Hemoglobins are important proteins known to act as NO storage or scavenger (Gupta et al., 2011). Based on their sequence homology and affinity for oxygen, three families of Hbs have been described in plants: Lbs, ns-Hbs, and truncated Hbs (tr-Hbs; Smagghe et al., 2009; Gupta et al., 2011). The three types of Hbs were reported to be expressed in legumes (Nagata et al., 2008; Bustos-Sanmamed et al., 2011). Lbs accumulate to millimolar concentration in the cytoplasm of infected nodule cells (Appleby, 1992). They are thought to buffer free oxygen in the nanomolar range, avoiding inactivation of nitrogenase while maintaining high oxygen flux for respiration (Ott et al., 2005). Deoxy-Lb was shown to bind NO with a high affinity to form stable complexes in soybean, and it has been proposed that Lb could act as a NO scavenger (Herold and Puppo, 2005). This may also be a function of the ns-Hbs which are ubiquitous in plants (Hill, 2012). Class 1 ns-Hbs could scavenge oxygen traces (Km # 2 nM) to convert NO to nitrate. They were suggested to be responsible for maintaining redox and energy status of plant cells under hypoxia (Igamberdiev and Hill, 2009). NO has been shown to up-regulate ns-Hb expression in a number of plant species. In the actinorhizal symbiosis between *Alnus firma* and *Frankia*, ns-Hb was strongly induced by the application of NO donors and it was shown that *Afns-Hb1*, as a NO scavenger, may support the N_2_ fixation ability of members of the genus *Frankia* (Sasakura et al., 2006). Similarly, the over-expression of *ns-Hb1* enhanced symbiotic N_2_ fixation in *Lotus japonicus* nodules (Shimoda et al., 2009). tr-Hbs were also shown to be induced in nodules of *M. truncatula* and *Datisca glomerata* (Vieweg et al., 2005; Pawlowski et al., 2007). Based on their expression pattern, it was proposed that they could be involved in NO scavenging. Three classes of Hb have been also described in bacteria: f-Hb (Hmp), single-domain Hb (sd-Hb), and tr-Hb (Sanchez et al., 2011). A bacterial strain of *S. meliloti* mutated in the f-Hb gene (*hmp*) elicited nodules on *M. truncatula* roots with higher levels of NO, lower N_2_ fixation efficiency and earlier nodule senescence than the WT (Cam et al., 2012), suggesting that the expression of the Hmp is essential for maintaining NO levels compatible with symbiosis even though plant Hbs are proficient. In *B. japonicum*, a sd-Hb was also shown to have a NO detoxification role under free-living, microaerobic conditions, suggesting that it could have similar role in nodules during NFS (Sanchez et al., 2011).

Beside Hbs, the respiratory Nor which catalyses reduction of NO into N_2_O, is also involved in NO degradation in rhizobia. Thus, in *B. japonicum* inoculated soybean plants subjected to flooding, a significant increase in NO and Lb-NO was observed in *norC* mutant compared with WT nodules (Sanchez et al., 2010). Similarly, NO level was increased in nodules of common bean exposed to nitrate, when elicited by a *R. etli* *norC* mutant as compared to the WT (Gomez-Hernandez et al., 2011). Interestingly, in *S. meliloti*, NO was found to induce *nor* expression (Meilhoc et al., 2010), and a *nor* mutant strain is more sensitive than a WT strain to a NO donor, and triggers early senescence of *M. truncatula* nodules (Meilhoc et al., 2013). It is important to note that Hmp does not compensate for the absence of Nor, and *vice versa*. On the whole, both plant and bacterial proteins participate in maintaining NO balance in nodules, and although the role of plant Hbs was underlined for years, bacterial NO-degrading enzymes should be considered as major components of this process.

## CONCLUSION AND FUTURE ISSUES

The data summarized in this review indicate that NO has dual effects in functioning nodules, inhibiting N_2_ fixation, on the one hand, and participating to energy metabolism, on the other hand. It may be considered as a regulator of N_2_-fixation and carbon metabolism, by inhibiting nitrogenase and/or enzymes of glycolysis and TCA cycle, to reduce energy demand in stress conditions such as a hypoxic environment. A challenging issue will be to assess precisely how much, where and when NO is produced inside the nodule. Regarding this point, the essential involvement of both plant and bacterial Hbs in the balance of NO level has been particularly evidenced, and much remains to be done to clarify the role of each of these proteins at tissue and cellular level in the functioning nodule.

Another promising issue will be to decipher the role of NO in the perception of oxygen under microoxic conditions. In mammals, a NO-dependent oxygen sensor system was identified, that works through a N-terminal mechanism for protein degradation which is activated by oxygen (Hu et al., 2005). Similar system was recently described in *Arabidopsis* plants, although its NO-dependence was not yet proved (Gibbs et al., 2011; Licausi et al., 2011). Functioning nodules, that are naturally microoxic but metabolically very active organs, appear to be an interesting model to analyze the functioning of such a system, and to investigate the interplay between low oxygen sensing, NO signaling, and metabolic regulation.

Crosstalk between reactive oxygen species (ROS) and NO appears to be a metabolic and signaling key to decipher symbiosis regulation. Peroxynitrite, which is formed when NO reacts with ${NO}_{2}^{-}$, is emerging as a potential signaling molecule to convey NO bioactivity by the selective nitration of Tyr residues in a small number of proteins (Vandelle and Delledonne, 2011). Since both NO and $O_{2}^{-•}$ are produced in symbiotic nodules (Puppo et al., 2013), it is conceivable that peroxynitrite is formed in these organs. Lb was shown to scavenge peroxynitrite, thus precluding any damaging effect of this species in the nodules (Herold and Puppo, 2005). The recent observation that glutamine synthetase GS1a is nitrated, whereas GS2a is subjected to *S*-nitrosylation in *M. truncatula* nodules (Melo et al., 2011), provides a direct link between NO/$O_{2}^{-•}$ signaling and N_2_ metabolism in root nodules. It may be also noted that many of the proteins identified as being *S*-nitrosylated in the symbiotic interaction have also been reported to be *S*-sulfenylated (Oger et al., 2012) suggesting that the same protein may be differentially regulated depending on redox state. The possible regulation of nodule NADPH oxidase activity by NO (Yun et al., 2011; Marino et al., 2012) could be important in the link between NO and ${NO}_{2}^{-}$.

## Conflict of Interest Statement

The authors declare that the research was conducted in the absence of any commercial or financial relationships that could be construed as a potential conflict of interest.
